# Incidence and Predictors of Gastrocutaneous Fistula in the Pediatric Patient

**DOI:** 10.5402/2011/686803

**Published:** 2010-12-01

**Authors:** Ioana Bratu, Aamir Bharmal

**Affiliations:** Pediatric General Surgery, Department of Surgery, Stollery Children's Hospital, University of Alberta, 2C3.56 WMC, 8440-112 Street, Edmonton, Alberta, Canada T6G 2B7

## Abstract

*Background/Purpose*. To determine the incidence, predictors, and outcomes of repair of gastrocutaneous fistulae (GCF) in pediatric patients. *Methods*. Patients were identified through a medical records search of all gastrostomy insertions performed from 1997–2007. *Results*. Of 1083 gastrostomies, 49 had GCF closure. Gastrostomy indications were reflux/aspiration (30/43 [70%]) and feeding intolerance/failure to thrive (7/43 [16%]). Gastrostomies were performed as open surgical procedures (84%) with fundoplication (66% of all cases) at an age of 0.5 ± 0.57 (median ± inter-quartile range) years. Gastrostomies were removed in outpatient settings when no longer used and were present for 2.3 ± 2.2 years, and GCF persisted for 2.0 ± 3.0 months. GCF were closed by laparotomy and stapling. GCF closure length of stay was 2.0 ± 3.3 days. Complications occurred in 6/49 patients and included infection/fever (4/6) and localized skin redness/breakdown (2/6). *Conclusions*. From our collected data, GCFs occur at a frequency of 4.5% and persist for 2.0 ± 3.0 months until closed. Given the complicated medical histories of patients and relatively high rate of postoperative infection/reaction (12.2%), GCF closure is not a benign, “uncomplicated” procedure. Further information describing factors determining which patients develop GCF requiring closure is needed.

## 1. Introduction

Gastrostomies provide enteral nutrition in children who require long-term nutritional support [1]. While minor and major postgastrostomy tube complications have been described [2–4], problems arising after gastrostomy tube removal have been less documented [5]. In some pediatric patients the communication between the stomach and the skin closes spontaneously, very quickly, in a matter of hours, but for some patients the communication persists for more than one month, becoming a gastrocutaneous fistula requiring surgical closure. We attempted to determine the incidence, predictors, and outcomes of repair of gastrocutaneous fistulas in pediatric patients.

## 2. Materials and Methods

All children age 0–17 years who underwent insertion of a gastrostomy tube over a ten-year period from 1997–2007 were reviewed. The study group included those children in whom a gastrostomy tube was subsequently removed and who required surgical closure of a persistent gastrocutaneous fistula. Patient and tube factors that might be predictive for the development of a persistent gastrocutaneous fistula were recorded: age, sex, primary diagnosis, method of initial gastrostomy insertion, length of time between insertion and removal, type of gastrostomy tube, French size of gastrostomy tube, method of gastrocutaneous fistula closure, and complications arising from gastrocutaneous fistula closure. 

Unfortunately as we only have complete medical information access to inpatient hospital charts, our study's limitation is that we could not make comparison between those children who had their gastrostomy tubes removed upon which the tract closed spontaneously and those who subsequently had persistent gastrocutaneous fistula tracts. Statistical analysis involved *χ* ^2^ analysis with Fisher's exact test. Significance was set at *P* < .05. 

## 3. Result

1083 patients had a gastrostomy tube placed during the ten-year period, of which 49 (4.5%) subsequently developed a persistent gastrocutaneous fistula requiring surgical closure. Original gastrostomy indications were gastroesophageal reflux disease (70%), feeding intolerance (16%), with the remaining cases being esophageal and swallowing dysfunction. 

At time of gastrostomy tube insertion, all patients had one of the following four comorbidities, either individually or in combination: respiratory tract infection predisposition with recurrent aspiration pneumonia, premature birth, congenital heart defect, or neurological deficit. Gastrostomies were performed as open surgical procedures in 84% of cases with fundoplication in 66% of all cases at a mean age of 0.5 ± 0.57 years (median ± interquartile range).

The most common size of the gastrostomy tube was 18 Fr malecot. The average length of stay following gastrostomy tube insertion was 8.0 ± 10 days reflecting the complicated nature of these patients. A third of patients had post-operative complications following gastrostomy insertion, including hospital readmissions or multiple clinic visits for review of their gastrostomy site. Complications included gastrostomy tube leakage, infection, or fundoplication review. 

**Figure 1:**
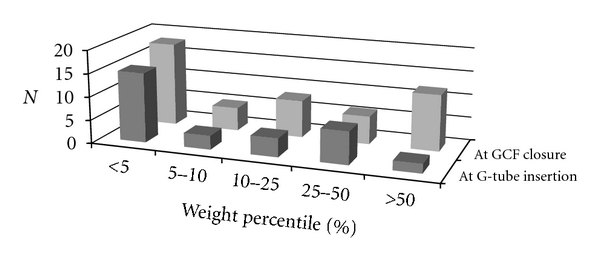
Age-matched weight percentiles of the patients with GCF at the time of original gastrostomy insertion and at GCF closure.

The weight percentile of children who had gastrocutaneous fistula closure was significantly greater (*P* =  .0097) than prior to gastrostomy tube insertion (Figure 1). 

Gastrostomy tubes were used on average for 2.3 ± 2.2 years and gastrocutaneous fistula persisted for 2.0 ± 3.0 months before surgical closure. Gastrostomy tubes were typically removed in outpatient settings when no longer needed, because of persistent leakage, or because of infection. All gastrocutaneous fistulae were closed by laparotomy and stapling. Length of stay for gastrocutaneous fistula closure was 2.0 ± 3.3 days. Post-operative complications following gastrocutaneous fistula closure occurred in 6/49 (12.2%) of patients and included infection/fever in 4 patients, and localized redness and breakdown of surgical site in 2 patients (Table 1).

Statistically significant correlations were observed between original age at gastrostomy insertion and subsequent admissions and complications for gastrostomy tube issues (*P* =  .004). Length of stay at the initial gastrostomy tube site insertion significantly correlated with a longer stay after gastrocutaneous fistula closure (*P* =  .038) indicating the complexity of these children. Moreover, developing a postoperative complication following gastrocutaneous fistula closure was significantly positively correlated with a longer length of stay after gastrocutaneous fistula closure (*P* < .001).

Gastrostomy tubes fulfill their primary function providing nutritional support, as these patients at the time of gastrocutaneous fistula closure were less likely to have failure to thrive. Patients who had long stays following gastrostomy tube insertions were more susceptible to postgastrocutaneous fistula closure complications and a longer hospital stay post gastrocutaneous fistula closure.

## 4. Discussion

Our data show a 4.5 % rate of persistent gastrocutaneous fistula requiring surgical closure. This is lower than the rate of 16%–45% reported in other studies [6–10]. This may partially be attributed to the retrospective nature of our study design where all cases of gastrocutaneous fistula requiring closure were identified through our medical records search as we do not have a database of all inserted gastrostomies where patients are tracked forward and complications recorded. It is thus difficult to know the exact reasons for our lower rate of fistula formation as most gastrostomies were done open with large malecots. While our reported rate of persistent gastrocutaneous fistula is lower than reported in the literature, it is important to remember that the prevalence in adults who have gastrostomy tubes upon which removal results in the persistence, is even lower. 

Children who require gastrostomy tube insertions and who subsequently develop gastrocutaneous fistula have complex medical histories. Surgical closure of gastrocutaneous fistula closure does carry associated risks with a high potential for post-operative complications, given the complicated nature of the patient population. While gastrostomies are needed for long-term enteral nutrition, especially in neurologically impaired children, it is important to keep in mind that gastrostomy insertion, and gastrostomy care thereafter does carry a relatively high rate of complications, and that complications can continue to occur even when the gastrostomy tube has been removed [1–10].

A small amount of the literature has attempted to determine predictive factors for persistent gastrocutaneous fistula in children [6–10]. The major determinant appears to be the length of time that the tube is in place prior to removal. It appears that all tubes removed within 8–11 months from initial insertion should be expected to close spontaneously. Patients within our data set who developed gastrocutaneous fistula had their gastrostomy tube in place for a longer period than 8–11 months, at a median length of 2.3 ± 2.2 years. 

The literature also suggests that children with previous renal failure who have had a renal transplant are also at higher risk for developing a persistent gastrocutaneous fistula. Other factors such as pre-existing medical illnesses including neurological disorders, gastrointestinal reflux, renal failure, steroid use, fundoplication presence, type of gastrostomy tube used, and type of surgical procedure to first place the gastrostomy used did not seem to predict gastrocutaneous fistula persistence. Like others, we also observed that nutritional status did not predict gastrocutaneous fistula persistence as most of these children demonstrated an increase in their percentile over time from initial gastrostomy tube insertion. 

While the standard method to repair gastrocutaneous fistulae is to close them surgically, novel, minimally invasive, approaches include endoscopic cautery, clip closure, endoscopic suturing, endoscopic assisted closure with a porcine fistula plug, endoscopic fibrin sealant injection with application of a haemostatic clip, or fibrin glue therapy for tract sclerosis [11–18].

Certainly it appears that time is of the essence and the longer the gastrostomy tube has been in, then, it is more likely for the resulting fistula to not close spontaneously. The risk of possible persistent gastrocutaneous fistula following gastrostomy removal should be communicated to parents at time of consent for the original gastrostomy insertion, realizing that surgical closure may be required in the future if the gastrostomy tube is no longer needed, as has been shown in 5%–45% of pediatric gastrostomy tube insertions. 

## Figures and Tables

**Table 1:** Descriptive of the patients undergoing GCF closure. Data is mean ± standard deviation.

Characteristics of patients with persistent gastrocutaneous fistula (GCF) *N* = 49	
Age	4.2 ± 3.6 years
Sex	13F : 36M
Duration of original Gtube	2.8 ± 2.3 years
Persistence of GCF until surgical correction	2.9 ± 3.4 months
Length of stay for GCF closure	3.1 ± 4.1 days
Complications after GCF closure	6 patients (skin/wound related)
